# Novel Approaches in Non-Melanoma Skin Cancers—A Focus on Hedgehog Pathway in Basal Cell Carcinoma (BCC)

**DOI:** 10.3390/cells11203210

**Published:** 2022-10-13

**Authors:** Paulina Chmiel, Martyna Kłosińska, Alicja Forma, Zuzanna Pelc, Katarzyna Gęca, Magdalena Skórzewska

**Affiliations:** 1 Department of Surgical Oncology, Medical University of Lublin, 20-081 Lublin, Poland; 2 Department of Forensic Medicine, Medical University of Lublin, 20-090 Lublin, Poland

**Keywords:** basal cell carcinoma, BCC, hedgehog inhibitors, HHI, patched homologue 1, PTCH1, Smoothened homologue, SMO

## Abstract

Basal cell carcinoma (BCC) is one of the most common neoplasms in the population. A good prognosis and mainly non-aggressive development have made it underdiagnosed and excluded from the statistics. Due to the availability of efficient surgical therapy, BCC is sometimes overlooked in the search for novel therapies. Most clinicians are unaware of its complicated pathogenesis or the availability of effective targeted therapy based on Hedgehog inhibitors (HHI) used in advanced or metastatic cases. Nevertheless, the concomitance and esthetic burden of this neoplasm are severe. As with other cancers, its pathogenesis is multifactorial and complicated with a network of dependencies. Although the tumour microenvironment (TME), genetic aberrations, and risk factors seem crucial in all skin cancers, in BCC they all have become accessible as therapeutic or prevention targets. The results of this review indicate that a central role in the development of BCC is played by the Hedgehog (Hh) signalling pathway. Two signalling molecules have been identified as the main culprits, namely Patched homologue 1 (PTCH1) and, less often, Smoothened homologue (SMO). Considering effective immunotherapy for other neoplastic growths being introduced, implementing immunotherapy in advanced BCC is pivotal and beneficial. Up to now, the US Food and Drug Administration (FDA) has approved two inhibitors of SMO for the treatment of advanced BCC. Sonidegib and vismodegib are registered based on their efficacy in clinical trials. However, despite this success, limitations might occur during the therapy, as some patients show resistance to these molecules. This review aims to summarize novel options of targeted therapies in BCC and debate the mechanisms and clinical implications of tumor resistance.

## 1. Introduction

The complexity of the skin is reflected in the variety of its neoplasms, both benign and malignant. The most popular division of skin cancers includes non-melanoma skin cancers (NMSCs) and melanoma. While focusing on NMSCs, we can distinguish two main types of cancer of epidermal origin; basal cell carcinoma (BCC) and squamous cell carcinoma (SCC) [1]. The incidence and mortality of NMSCs are not fully known due to limited data from unreported cases. GLOBOCAN estimated the incidence of skin cancers in 2020 to be over a million new cases, excluding BCC as one of the most common oncological pathologies. Mortality statistics included BCC, reaching 63,000 in 2020 [2].

BCC may cause a significant impairment of organ functions, deformation, lowering the standard of living [3]. Due to its low metastatic potential, local surgical treatment is the treatment of choice [3,4,5]. Some patients, especially the elderly with BCC localized on the face, may benefit from radiotherapy because of the limited usage of surgical treatment within the face [4]. An excellent prognosis is observed with this approach [6,7]. Moreover, in 2021 FDA approved cemiplimab, a monoclonal IgG anti-PD-1 antibody, for treating patients with locally advanced and unresectable BCC [8].

Among many aberrations observed in BCC, dysregulation of the Hedgehog (Hh) signalling pathway is the most characteristic and typical. The Hh pathway mostly depends on inactivating mutations in a negative regulator, *PTCH1*, or less often on activating mutations in a positive regulator, *SMO*. Aberrations in *PTCH*1 as a classic tumour suppressor gene affect SMO activating functions, which leads to activation of glioma-associated oncogene 1 and 2 (*GLI1/2*) [9,10]. Moreover, mutations in the *SMO* proto-oncogene may show the same effect on cell functions [11]. Activating the Hh pathway in keratocytes initiates the decrease of control over differentiation and proliferation, which is necessary to maintain cell populations and regulate the development of sebaceous glands and hair follicles (10). FDA approved systemic Hedgehog inhibitors (HHIs) in cases of locally advanced BCC (laBCC) or metastatic BCC (mBCC) [12]. However, overcoming resistance to HHIs remains a crucial research topic [13,14,15,16]. The aim of this review is to summarize the latest literature regarding BCC, the concomitant dysregulation in the Hh signalling pathway, as well as to generate discussions on paths toward the future study and treatment of BCC.

## 2. Non-Melanoma Skin Cancers—An Overview

Skin cancers are the most common malignancies worldwide, with the most prevalent being BCC, SCC and melanoma [17]. The general classification highlights four disease types: epidermal, benign, malignant and melanocytic benign, or malignant. BCC and SCC fall into the malignant epidermal category [1]. It has recently been observed that the incidence of skin cancers is increasing; however, the data is unclear and dependent on the geological region [18,19]. BCC and SCC represent the majority of NMSCs [5]. In the USA, the incidence rates of NMSCs were measured from 1990 to 2019. The incidence increased from 402 to 787 per 100,000 persons, yet the mortality remained relatively stable with an estimation of 0.8 [6].

**Figure 1:**
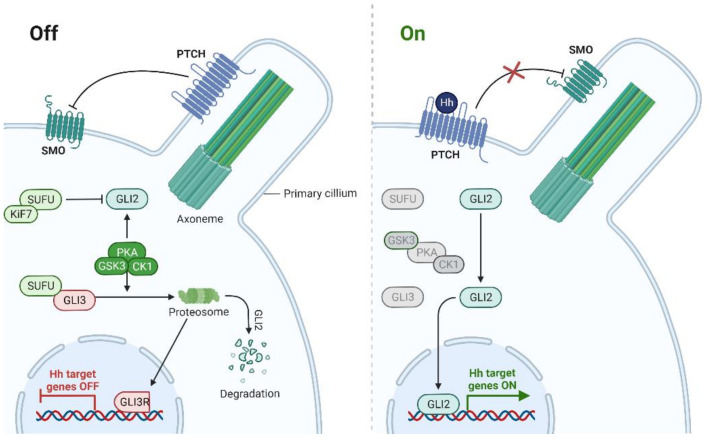
Outlook on primary cilia and Hedgehog signalling pathway [87]. In the absence of a Hh ligand, GLI is phosphorylated by PKA, GSK3β, and CK1, leading to GLI repressor formation and arrest of the pathway. When Hh ligands are present, Smoothened is phosphorylated, SUFU inhibition is removed, and the GLI activator induces targeted genes transcription.

The development of skin cancers is conditioned by multifactorial mechanisms, including genetic mutations, phenotypical characteristics, and environmental factors [20]. With the highest incidence in Caucasian people, one of the leading causes of NMSCs is exposure to ultraviolet radiation [21,22]. UV light is proven to induce damage to cellular DNA and RNA directly, causing the formation of covalent bonds between adjacent pyrimidines (UVB) as well as producing reactive oxygen species (UVA) [23]. Sun exposure, sunbed use and phototherapy pose risks associated with high UV radiation. Furthermore, fair skin type, male sex, immunosuppression, transplant reception, radiotherapy, and arsenic exposure are the risk factors of carcinogenesis (Figure 1) [24,25,26]. A common genetic mutation related to an increased BCC prevalence regards nevoid BCC syndrome with germline mutations of *PTCH1*, a tumour suppressor and Hh receptor. Consequently, this results in the decreased suppression of intracellular signalling by the G-protein-coupled receptor, SMO, which leads to the direct upregulation of target genes’ transcription [27]. Somatic *PTCH1* mutations often present in familial and sporadic BCC [28,29]. Another common dysregulation regards *TP53* tumour suppressor gene inactivation by UV radiation in many cases of BCC [30,31]. Genes also involved in NMSCs pathogenesis are melanocortin-1 receptor (*MC1R*), xeroderma pigmentosum complementation group C (*XPC*), cytochrome P450 2D6 (*CYP2D6*), cyclin dependent kinase inhibitor 2A (*CDKN2A*), glutathione S-transferase theta 1 gene (*GSTT1*), and telomerase [20,23].

## 3. Basal Cell Carcinoma

BCC is a malignant neoplasm, representing about 80% of NMSCs, and typically occurs without precursor lesions compared to SCC [32,33]. Many histological subtypes of BCC can be distinguished, as they come with different clinical manifestations. Nodular BCC is the most common one and accounts for almost 60% of BCCs. It develops as raised pink pearly nodules with surface telangiectasia and ulceration [20]. Superficial BCC is less frequently diagnosed and is mainly located on the trunk of sun-protected areas and limbs. Superficial BCC manifests as pink macules or thin plaques, and can be easily mistaken for other dermatological diseases [20]. Moreover, we can distinguish morphoeic BCC, generally located on the nose and appearing as scar-like plaques [20,34]. It can also cause extensive local destruction [3]. However, they are both more aggressive than nodular BCC [35,36]. Most BCCs occur in the head and neck area with the exclusion of superficial BCC [36]. Accompanying symptoms that allow the diagnosis include crusting, bleeding, tenderness, and itching [33]. Although metastasis is rarely observed, local growth tends to be destructive [27]. Dermatoscopy examines and identifies arborising telangiectasia, ulceration, and leaflike areas characteristic of BCC [37]. Skin biopsy enables the exact BCC subtype identification, while computed tomography or magnetic resonance imaging can play a pivotal role in the diagnosing of bony, vascular, or major nerve invasion [23].

BCC’s preferred therapeutic approach depends on low- or high-risk assessment. Surgical excision remains the most common one, with a particular significance of Moh’s micrographic surgery, which allows a better histological accuracy of complete tumour resection with maximized tissue conservation [38]. This method is the most frequently chosen and highly effective in the case of facial BCC [39]. Destructive techniques are highly successful in BCC treatment, including radiotherapy, photodynamic therapy, topical imiquimod therapy, cryosurgery, curettage and cautery [24,40,41,42,43]. Moreover, immunotherapy with programmed cell death PD-1 antibodies is currently undergoing investigation in clinical trials and, as mentioned before, cemiplimab has been approved by the FDA for treatment of advanced BCC [4,8] The main focus in search for efficient treatment is on Hedgehog Pathway Inhibitors, as they have become a new target in therapy designated for BCCs not qualified for surgery or radiotherapy [44]. Vismodegib and Sonidegib have also proved to be effective in treating advanced and metastatic forms of BCC [13,14].

## 4. Hedgehog Pathway and Dysregulation in Malignancy

### 4.1. Hh Pathway Overview

The hedgehog pathway is also known as Hedgehog-Patched (Hh-Ptch), Hedgehog-Gli (Hh-Gli), or Hedgehog-Patched-Smoothened (Hh-Ptch-Smo). It is vital in the early stages of embryonic development during vertebrates and invertebrates shaping and renal development [45,46]. In adult organisms, it is rarely activated, mainly during wound healing and in pluripotential cells crucial for tissue repair [47,48,49,50,51,52]. The hedgehog signalling pathway is the most characteristic for primary cilia (PC), a structure in charge of chemical, thermal and mechanical signalling [53]. Studies indicate that Hh pathway physiological functions can enhance the development of multiple malignancies by promoting metastasis and proliferation and dysregulating TME [54,55,56]. Currently, there are two known ways of activating the Hh pathway—canonical with ligand or receptor interaction and non-canonical downstream pathway, SMO independent [57]. Three proteins are crucial in signaling; Hh ligand, PTCH, and SMO transducing the signal into activation of GLI transcription factors [58]. PTCH, a receptor in the Hedgehog signalling pathway, is a 1500 amino acid protein that spans the cell membranes 12 times with two extracellular loops binding the Hh ligand [59]. Both the N- and C-terminal domains of PTCH are cytoplasmic [60]. As for now, two major PTCH receptors are distinguished; PTCH1 and PTCH2, the first playing a vital role in most pathologies [59]. The absence of the Hh ligand causes the PTCH derived arrest of SMO translocation. The lack of this factor enables protein kinase A (PKA), glycogen synthase kinase-3 (GSK3), and casein kinase 1 (CK1) to phosphorylate full-length glioma-associated oncogene (GLIFL) and create a Gli repressor (GLIR) (Figure 1) [61,62]. GLIR is responsible for repressing the expression of the Hh signalling pathway target genes [63,64]. Three genes are found to be in charge of activating the Hedgehog pathway through their products; the Sonic Hedgehog (*SHH*), Indian Hedgehog (*IHH*), and Desert Hedgehog (*DHH*) [65]. Their expression depends on the tissue type [65]. The signaling pathway starts with linking any of the ligands (Hh) with PTCH protein, so the complex is internalized. The blocked inhibition of SMO enables the signal to go through numerous cytoplasmic proteins; kinesin protein (KIF7), suppressor of fused (SUFU), and GLIFL [66]. Eventually, the GLI activator (GLIA) is released and activates the transcription of specific genes through Gli transcription factors [57,67]. GLI1, GLI2, and GLI3 are factors primarily discovered in glioblastoma and members of the Kruppel family of zinc-finger transcription factors. However, each plays a different role in pathway signaling [68,69]. GLI1 acts as a transcription activator, GLI2 has a dualistic role, while GLI3 is correlated with repression of transcription [70,71,72]. In addition, GLI factors can be controlled by the SUFU, when in the absence of SHH it binds directly to GLI and represses its activation [73,74]. The Hh pathway focuses on multiple genes involved in cell functioning as well as genes that regulate the Hh pathway such as *PTCH1* and *GLI1* (Table 1) [75,76]. Numerous studies have established a connection between the Hh pathway and other crucial kinases often linked to carcinogenesis and targeted in anti-tumour therapy. Kaesler et al. found that PKA can inhibit GLI1 transcriptional activity, while Wang et al. showed that the arrest of GLI dependent on PKA regulation can result in limb malformations [77,78]. Dual specificity Yak1-related kinase 1 (DYRK1) could also control the transcriptional activity of *GLI1* in a not fully understood way [79]. Riobo et al. identified that both protein kinase C-delta (PKC-δ) and mitogen-activated protein/extracellular signal-regulated kinase-1 (MEK-1) are positive regulators of the Hh pathway through GLI activity [80]. The same authors presented the crucial influence of phosphoinositide 3-kinase (PI3-kinase)-dependent AKT on the Hh signaling pathway in signal transmission. Stimulating PI3-kinase/Akt by insulin-like growth factor I (IGF-1) provides GLI activation even with low levels of Hh [81]. Finally, the Hedgehog signalling pathway has its own internal negative feedback system, which depends on GLI2 and GLI3 activation [82,83,84]. If the Hh ligand is absent, SUFU phosphorylates many residues in order to cleave GLI2 and GLI3 into smaller proteins with the repressor function, which allows negative feedback to go through. However, out of these two proteins, GLI3R is known to be more stable and translocate to the nucleus where it inhibits Hh-related responses, with GLI2R marginal function in inhibition [85]. In GLI independent, the non-canonical pathway signalling is divided into two mechanisms. The first one is dependent on PTCH1 and Hh ligands without the use of SMO, and it can be mainly applied to cell proliferation. The second type goes through SMO, affecting calcium ions, chemotaxis, and cell migration, while promoting cell proliferation and survival depending on the Src kinase family [86].

### 4.2. Hedgehog Links with Other Crucial Pathways

The correlations of the Hh pathway with other well-known pathways of carcinogenesis are also possible targets worth investigating. Interactions with other signalling components, such as TGF-β, EGFR, KRAs, PKA, NOTCH, and Wnt/β-catenin, were spotted. Several of these signals can be active in one malignancy [102]. Maeda et al. showed that beta-catenin could be involved in Hh-signalling through the enhancement of the transcriptional activity of GLI [103]. SUFU, on the contrary, can suppress the activity of beta-catenin and functions as a negative regulator of T-cell factor (Tcf)-dependent transcription [104]. Studies carried out on medulloblastoma with mutant SUFU showed that the aberrations of this protein can stimulate both the Wnt and Hh pathways, however research on BCC is still needed [105]. The results of a few studies suggested that the EGFR oncogenic pathway has a synergistic effect on cancer cell proliferation and survival, whereas simultaneous overexpression of GLI1 and MEK1 induces tumour development in studies with BCC [106]. Götschel et al. concluded that the EGFR signalling silences proteins acting as negative regulators of Hh signalling, and conversely, Hh signalling keeps the strong and significant expression of numerous canonical EGF-targets [107]. All that evidence encourages expanding the search for therapeutic options with combined therapy targeting multiple oncogenic proteins at once for better results in patients.

This complexity of Hh pathway signalling and the multitude of connections with alternative ways of tumour development indicate both pathogenesis and possible treatment aims in solid tumours, one of which is BCC. The relationship between the Hh pathway and human skin pathology was discovered for the first time in a rare disease, namely, the nevoid basal cell carcinoma syndrome (NBCCS). NBCCS, known as Gorlin syndrome, is an autosomal dominant disorder characterised by multiple BCCs, basal cell nevus on palms and soles, jaw keratocysts, and various other tumours [108,109]. The basis for the development of this syndrome is a mutation in the *PTCH1* gene located on chromosome 9q22.3. Moreover, the loss of the heterozygosity of this region is recognized as elementary for the development of sporadic BCC [110,111]. Studies indicated that aberrations in Hh signalling are present in around 90% of these malignancies [10]. Most common aberrations lead to the activation of the Hh pathway irrespective of the presence of the ligand, precisely the activating mutations in the *SMO* or inactivating mutations in the *PTCH1* or *SUFU* [29]. Many preclinical models and studies support this thesis, as approximately 85% of sporadic BCCs have the inactivating mutation of *PTCH1,* and in UV-inducted in the *PTCH1* mutant mice population*,* BCC incidence was spotted [112,113]. However, sporadic BCC can also occur in the overexpression of GLI2, regardless of upregulation mechanisms [114]. At the same time, blocking the signalling pathway in genetically modified mice allowed for the involution and reduction of the proliferation of BCC cancer cells [115]. With all clinical and therapeutic implications, BCC remains one of the most closely correlated with Hh signalling neoplasms.

### 4.3. Interactions between Hh and TME

Furthermore, a strict correlation between TME and the Hedgehog pathway was spotted. The Hh pathway can transduce signals in autocrine, juxtacrine, and, moreover, a paracrine manner; which influences various TME cells [116]. Studies highlight the importance of targeting TME in anti-tumour therapy as one of the main alterations promoting tumour progression and development. Lack of immune control and overpopulation of disrupted malignant cells leads to cancer development. Models have shown that the Hedgehog pathway is downregulated in multiple inflammatory diseases, and pathway activation can be linked with an anti-inflammatory effect [117,118]. Hh signalling can be strictly involved in the immunological activity of cells in TME by remodeling the microenvironment (104). Tolerance in the microenvironment can be disrupted by the expansion of CD4+CD25+FoxP3+ regulatory T-cells (Tregs) that are primarily dependent on PD-L1 overexpression induced by the Hh pathway [119]. Furthermore, inflammatory tumour-infiltrating monocytes (TAMs) stimulate the progression of pancreatic cancer by expressing Hh pathway genes [120].

Hypoxia, a critical factor in the expression of multiple immune checkpoint proteins, is accountable for the overexpression of PD-L1 and the activation of the Hedgehog pathway. However, Hh inhibitors can reduce PD-L1 expression in cancer cells [121,122]. The activation of hypoxia-inducible factor-1α (HIF-1α) by tumour hypoxia strongly activates the secretion of the SHH ligand by cancer cells, which promotes proliferation and tumour survival [123]. The anti-tumour response can be conducted with IL-4, IL-5, IL-6, IL-10, and IL-13, which are higher in BCCs with an activated Hh pathway [124]. Additionally, significant levels of PD-L1, PD-L2, TIGIT, TIM3, and CD226 were reported in BCC-like skin tumours in which the TME is enriched in T-cell populations with Hh pathway activation [124]. Geng et al. showed that the activation of Hh caused increased angiogenesis in the TME, whereas the vascular network was reduced using the SHH inhibitor [125]. Considering the growing resistance to SHH inhibitors, the strong correlation of this signalling pathway with targetable immune checkpoints creates new perspectives for the combined therapy of tumours such as BCC.

## 5. Hedgehog Inhibitors in Non-Melanoma Skin Cancers

The treatment goals include achieving the best clinical outcome with prevention of function and long-lasting cosmetic effects. In advanced or metastatic disease, surgical treatment does not provide radical excision, so its implementation is limited in these cases. As indicated, targeting the Hedgehog pathway significantly affects BCC progression and patient outcomes. Since many elements may interrelate in the Hh pathway, various small molecules are a matter of research [60,126,127]. Depending on the targeting agent, they can be divided into groups: SHH inhibitors, SMO antagonists, and GLI inhibitors. The two most well-established are sonidegib and vismodegib, the only registered oral agents for metastatic or advanced BCC.

### 5.1. SHH Inhibitors

So far, four agents have been described and are currently being investigated: robotnikinin, RS-U 43, the 5E1 monoclonal antibody, and 7_3d3. All of these studies are in the pre-clinical stage and are providing promising results. Robotnikinin is a small molecule targeting Sonic hedgehog through six amino acids and the Zn(II) ion present in the binding groove of SHH [126]. It appears as an excellent therapeutic option for patients with elevated SHH ligand expression [128,129]. RS-U43 is a dihydrothienopyridine derivative that blocks SHH palmitoylation—a key factor in Hh signalling. This action effectively inhibits autocrine and paracrine SHH signalling [130]. The 5E1 monoclonal antibody acts by blocking the interaction of SHH with PTCH1, resulting in an error in pathway signalling [131]. Lastly, 7_3d3 is a derivative of pyrimidine, whose activity, when measured by IC50, showed that, after modification, it is a molecule ten times stronger than robotnikinin, reaching 0.4 μM in IC50 value. The ability of 7_3d3 to interact with Hh has been shown in vitro [132].

### 5.2. SMO Antagonists

The most widely studied group of compounds and the subject of the most intensive clinical trials in BCC are SMO antagonists. They bind pockets within the extracellular or transmembrane domain of SMO [133,134]. The first tested antagonist was cyclopamine, a natural steroidal alkaloid derived from *Veratrum californicum*. Preclinical studies indicated that the molecule inhibits tumour growth and can induce the death of cancer cells. Consequently, the first clinical trials with this agent confirmed its safety and efficacy in recurrent BCC after surgical excision [135,136]. However, in successful clinical trials, cyclopamine was administrated topically, since when administered orally in animal trials, there were significant side effects, including dehydration and death. It was also poorly absorbed after oral administration [137]. All of these effects limit the clinical use of cyclopamine.

Vismodegib and sonidegib are the most known agents of this group. A study that enabled vismodegib registration—ERIVANCE—was conducted on patients with pathologically confirmed, recurrent laBCC or mBCC. The results showed that the overall response rate (ORR) was 30.3% in 33 patients with mBCC and 42.9 in 63 patients with laBCC; the median response duration was 7.6 months. The most common AEs were muscle spasms, alopecia, dysgeusia, weight loss, atrial fibrillation, hypocalcemia, and hyponatremia [127]. At the same time, the ERIVANCE study provided a comprehensive safety profile of vismodegib, which showed poor results, as 25% of patients had serious AEs and seven AE correlated death was spotted [138]. Since then, multiple trials have begun, the two main being STEVIE and MIKIE. The primary endpoints of the STEVIE trial were the occurrence of AEs during oral therapy with 150 mg of vismodegib. 98% had ≥1 treatment-emergent adverse event (TEAE), yet they were not severe enough to exclude patients from the trial. The safety profile matched previous studies [139]. Because a majority of patients to whom vismodegib was administered experienced AEs, the MIKIE study was carried out, with different, intermittent treatment schedule of the molecule which allowed to lower a single dose. The MIKIE study’s primary endpoint was a percentage reduction from baseline in the number of clinically evident BCCs. Patients were divided into two groups which differed in treatment schedule, and the reduction in group A totaled 62.7%; while in group B it totaled 54.0% [140]. The first combined treatment was applied to patients with vismodegib and pembrolizumab. The primary outcome was the ORR in both arms of the study, and secondary outcome measures included the incidence and severity of AEs [141]. In 2015, based on the BOLT trial, the FDA approved sonidegib for patients with laBCC and mBCC. Patients were randomized into two groups to receive sonidegib at 200 mg or 800 mg daily. The primary endpoint was ORR, and secondary endpoints were duration of response (DoR), CR rate, time to tumour response (TTR), and progression-free survival (PFS). The ORR in laBCC patients was 47.0% in the 200 mg group and 35.2% in the 800 mg group. Patients with mBCC achieved 15.4% in the 200 mg group and 17.4% in the 800 mg group. Safety was evaluated, and the most common AEs were muscle spasms, dysgeusia, weight decrease, and nausea. Sonidegib showed efficacy and safety for advanced and metastatic BCC with a more favourable benefit-risk profile for 200 mg [142]. Follow-ups were performed after 30 and 42 months of the BOLT study, mainly demonstrating the long-term efficacy and safety of the 200 mg dose. ORR was sustained and finally reached 56% in laBCC and 8% in mBCC. Furthermore, sonidegib showed a better safety profile than vismodegib [14,143]. Several other Smo inhibitors such as glasdegib, SANT-1, CUR-61414, and ALLO1/2 are currently in preliminary studies, with a need for further investigation [144,145,146,147].

Currently, novel agents are evaluated in clinical trials. TAK-441, an oral antagonist, was tested in a phase 1 study with patients with advanced non-haematological malignancies, with 21% of patients diagnosed with BCC. The observed inhibition of GLI and the lowering of its levels depending on dose resulted in a partial response (PR) in one patient and stable disease (SD) in seven patients [148]. XL139, currently in phase 1 study, is administrated orally to patients with advanced BCC. The agent showed its activity towards inhibiting Smo [149,150]. LEQ506 is another oral inhibitor in the dose-escalation study. The aim of the novel phase 2 study on taladegib, currently awaiting recruitment, is to evaluate the efficacy and safety in patients with loss of function of PTCH1 and advanced solid tumours based on occurring adverse events (AEs) and Response Evaluation Criteria in Solid Tumours (RECIST1.1) [151]. There are a few trials with Patidegib; a topical gel indicated for patients with sporadic BCC and Gorlin syndrome. All these studies have proved that administering patidegib topically guarantees the safety and efficacy of therapy with no systemic AEs [152]. At the same time, a decreased amount of *GLI1* mRNA transcript was observed [152].

### 5.3. Gli Inhibitors

In light of growing resistance and indicating new activation methods of the Hh pathways, targeting GLI1 and GLI2 as eventually executive units seems crucial. Lauth et al. proposed two agents blocking GLI proteins from interfering with their DNA binding capacity. These are GANT58, which is capable of inhibiting the activity of GLI1, and GANT61, inhibiting both GLI1 and GLI2 [153]. ATO, another GLI inhibitor, is currently approved for the treatment of acute leukemia [154]. It manipulates the action of GLI in multiple ways, such as by increasing degradation and directly binding to GLI proteins [155,156]. Moreover, and which will be described in further detail, combined therapy with ATO and itraconazole proved to overcome acquired resistance to SMO antagonists in BCC [157]. Small molecule Hh pathway inhibitors (HPI-1/2/3/4) showed the ability to block the pathway in numerous ways, mainly by inhibiting GLI. The direct mechanisms of their activity remain unknown. While HPI-1 can suppress Hh pathway activation induced by the loss of SUFU or GLI overexpression, HPI-2 is mostly effective towards GLI2, and HPI-4 disrupts ciliogenesis. The most essential features of HPIs are acting downstream to SMO and their ability to interact with posttranslational modifications [158].

## 6. Resistance in Studies and Clinical Practice

Two resistance models are distinguishable during treatment with HHI. There are patients with advanced BCC who did not respond to any HHI in the initial treatment (primary/intrinsic resistance), and patients who relapsed after an initial response to HHI treatment (acquired resistance). The first case of resistance occurred in 2009 when Rudin et al. described a patient with medulloblastoma resistant to HHI [159]. After that, many cases of resistance were reported. Even in clinical trials where FDA approval was granted, some patients lacked response or developed resistance during treatment. In ERIVANCE follow-up after 39 months from 104 enrolled patients, 27.9% had progression of the disease [13]. At the same time, disease progression was the most common reason for patient withdrawal from the ERIVANCE trial [138]. Likewise, trials with sonidegib showed PD in some patients; 29.1% of patients receiving sonidegib 200 mg and 9.9% of patients receiving sonidegib 800 mg had PD [143]. Numerous cases pointed to primary resistance for HHIs in spontaneous BCC and Gorlin syndrome [15,16,160]. After a complete response, acquired resistance mechanisms occur, leading to the recurrence of malignant processes and metastases within a few months [161]. An exploratory open-label study was conducted to evaluate the safety of sonidegib combined with buparlisib for BCCs that did not respond to prior treatment. In fact, four of the seven enrolled patients had SD [162]. Cross-resistance was observed between sonidegib and vismodegib [163]. That being said, a conclusion can be drawn that targeting multiple pathways involved in BCC pathogenesis seems beneficial for patients with resistance, as buparlisib is PI3K specific inhibitor. Moreover, Kong et al. described a sensitizing effect of buparlisib in combination with chemotherapy [164].

**Figure 2:**
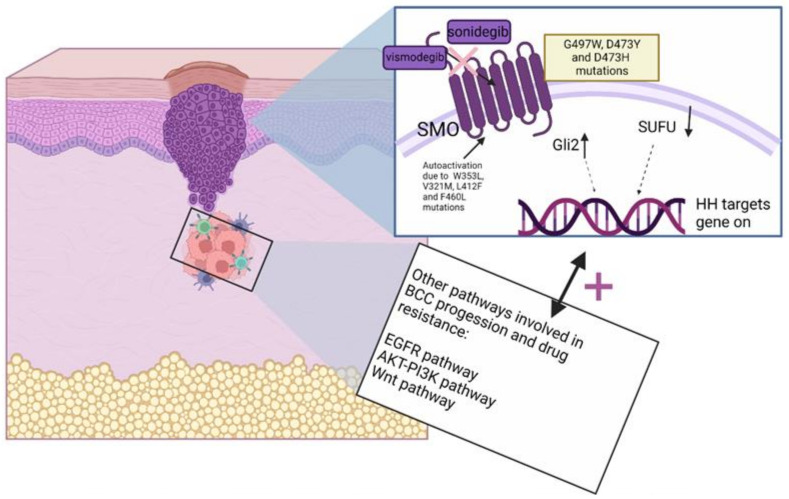
The most common resistance mechanisms in Hedgehog inhibitors treatment involving canonical and non-canonical ways of activating the Hh pathway.

Currently, several resistance mechanisms are described in the literature (Figure 2). *SMO* mutations and a binding site for HHI are the most common causes of resistance both in BCC and other Hh-dependent neoplasms, mainly medulloblastoma [165,166]. However, the mechanisms of resistance appear to be similar in tumours with upregulation of the Hh pathway. In intrinsic and acquired resistance, most patients carry the *SMO* mutation [167,168]. Mutations, namely p.G497W, p.D473Y, p.D473H and the most frequent p.D473 and p.W535L, may result in the ongoing activity of the Hh pathway even in the presence of various inhibitors, as shown in medulloblastoma [169]. Molecular screening of tumour specimens showed that the p.D473Y mutation directly causes vismodegib binding affinity, whereas p.G497W mutation is known to interfere with drug entry to the binding site [168]. The P.D473Y mutation can be linked with resistance for both sonidegib and vismodegib in medulloblastoma and can be used as a negative predictive marker in the future [170]. Interestingly, a genomic analysis proved that most cases of recurrent BCC harbored mutations in *SMO*, when only 15% of previously untreated neoplasms showed these alterations [167]. Another less commonly used preparation, itraconazole, has been tested in resistant BCC. An antifungal agent, itraconazole, has shown efficiency as a potent Hh pathway inhibitor in medulloblastoma [171]. Simulations showed that it is possible for itraconazole to bind different sites of SMO: the pocket in the C-terminal domain instead of the N-terminal domain, and the binding does not interfere with vismodegib binding, yet this evidence was not documented in experimental trials [172]. At the same time, itraconazole effectively reduced lesions and inhibited disease progression in patients with BCC [173]. Other resistance mechanisms concerning SMO mutations can block the autoinhibitory impact of the SMO loop structure. Mutations such as p.W353L, p.V321M, p.L412F, and p.F460L may cause constant activation of the Hh pathway [174]. Genetic alterations outside of *SMO* were found in resistant BCCs, such as reduced of *SUFU* and increased copy numbers of *GLI2* [167]. The non-canonical way of activating GLI contributes to resistance by upregulation of other oncogenic pathways. Due to the tumor’s heterogenic character, canonical and non-canonical pathways may co-exist in a given cancer type [175]. Other less common resistance mechanisms involve the association of Hh with other cell signalling pathways and switching between canonical and non-canonical activation. Indeed, Whitson et al. described a noncanonical hedgehog activation pathway driven by the transcription factor serum response factor (SRF) and its coactivator megakaryoblastic leukaemia 1 (MKL1) [176]. Moreover, Biehs et al. suggest tumour cell identity switching by activating the Wnt pathway, which allows the tumour to survive during vismodegib treatment [177]. Furthermore, a study conducted by Sanchez-Danes et al. showed that in BCC resistance to therapy may occur through a small population of cells with upregulated Wnt signalling, which persist the standard therapy of vismodegib [178]. Dheeraj et al. described a decreased level of phosphorylated EGFR and AKT in HHI resistant, previously treated BCC [179]. In conclusion, numerous cases of resistance occurred; however, their mechanisms are not fully understood and are often linked to medulloblastoma or different neoplasms, which does not always correlate with BCC. Future, in depth research is needed as overcoming resistance is crucial for efficient dosage, reduction of AEs, and toxicities linked with molecular targeted treatments.

## 7. Conclusions and Future Directions

The occurrence of BCC is primarily associated with the mutations that lead to the upregulation of the Hedgehog signalling pathway. Thus, researchers continuously strive to develop such inhibitors and targeted therapies that would specifically inhibit the carcinogenic effects of the disturbed Hedgehog pathway. Several drugs are already approved, while other therapies are still undergoing investigation. The major concern occurring in clinical trials and pre-clinical studies is the limited usage of novel compounds. Many of the discussed molecules have been investigated only in models, pre-clinical studies and on limited groups of patients. Considering current interventions, the usage of SMO inhibitors seems to be beneficial. Nevertheless, possibilities and multitudes of new targets and drugs should be effectively used and investigated. However, a significant potential side effect in the form of the induction of SCC should also be considered. Drugs such as GLI antagonists, which aim to inhibit the bromo and extraterminal (BET) domain family, also present good clinical outcomes regarding BCC treatment. Despite high clinical efficacy, severe adverse effects of specific Hedgehog pathway-targeted therapies should be considered. Another issue constitutes the molecular characterisation of a patient’s BCC to introduce the most effective therapeutic approach. Furthermore, some tumours might develop drug resistance during therapy, significantly decreasing the overall clinical outcome with the necessity of implementing other treatment strategies. For this reason, there is a need for other drugs to be developed and investigated in clinical trials. Combined therapies are proposed for the patients to minimise potential side effects and effectively manage the neoplastic growth of BCC. Additional modalities include immunotherapy or photodynamic therapy. A significant aspect of novel BCC-focused therapies is the search for other mutations beyond those related to the Hedgehog signalling pathway. The breakthrough would allow the development of therapies that could inhibit the major carcinogenic pathway and facilitate BCC growth progression. Combined treatment modalities targeting various signaling pathways of BCC (and not only the Hedgehog pathway) could constitute an opportunity to treat advanced, metastatic, and recurrent BCC that is resistant to treatment. In conclusion, the approval and common usage of the drugs that target the Hedgehog pathway constitutes an important treatment modality for treating BCC patients. However, alternative drugs, particularly resistant tumours, should be investigated.

## Figures and Tables

**Table 1:** Hh signalling pathway related and targeted genes, the most crucial in oncogenesis and signalling.

Gene	Effect of the Gene Product in Carcinogenesis	Reference
*PTCH1*	Regulator of SMO, negative feedback	[76]
*PTCH2*	Regulation of SMO, negative pathway feedback	[76,88]
*SMO*	Signal transducer, activator of GLI transcriptional factors	[89]
*SUFU*	Negative regulator of Hh signalling pathway	[90]
*GLI1*	Positive pathway feedback	[76]
*HHAT (*Hedgehog acyltransferase*)*	Catalyzation of the covalent attachment of palmitate, activation of the downstream signalling	[91]
*HHIP* (Hedgehog interacting protein)	Controlling of Hh pathway with negative feedback, growth, migration, and invasion of cancer cells	[92,93]
*GAS1*	co-receptor, activation of PTCH2-dependent Hh signalling	[94]
*CCND2*	Promotion of cellular growth, induction of DNA replication	[95]
*CCNE1*	Promotion of cellular growth, induction of DNA replication	[95]
*BCL2*	Regulation of apoptosis, inducing malignant phenotype	[96]
*MYCN*	Induction of Hh-induced proliferation, promotion of cell cycle entry	[97]
*PAX6/7/9*	Phenotypic transformation, proliferation, and migration via Hh signalling pathway	[98]
*JAG1*	Homeostasis of stem and progenitor cells, involved in Wnt signalling pathway	[99]
*WNT2B*	Cancer progression, key factor in Wnt signalling pathway	[100]
*FOXM1*	Regulating the expression of genes involved in cell growth, proliferation, differentiation, longevity, and transformation	[101]
